# Association of *CDKN2BAS* Polymorphism rs4977574 with Coronary Heart Disease: A Case-Control Study and a Meta-Analysis

**DOI:** 10.3390/ijms151017478

**Published:** 2014-09-29

**Authors:** Yi Huang, Huadan Ye, Qingxiao Hong, Xuting Xu, Danjie Jiang, Limin Xu, Dongjun Dai, Jie Sun, Xiang Gao, Shiwei Duan

**Affiliations:** 1 Department of Neurosurgery, Ningbo First Hospital, Ningbo 315010, China; E-Mails: huangy102@gmail.com (Y.H.); nbyysj@sina.com (J.S.); 2 Zhejiang Provincial Key Laboratory of Pathophysiology, School of Medicine, Ningbo University, Ningbo 315211, China; E-Mails: yehuadan@163.com (H.Y.); hongxiao9002@163.com (Q.H.); xuxuting1989@gmail.com (X.X.); jdj0526@yeah.net (D.J.); wendy880803@163.com (L.X.); daidongj789@126.com (D.D.)

**Keywords:** coronary heart disease, single nucleotide polymorphism (SNP), *CDKN2BAS*, meta–analysis

## Abstract

The goal of our study was to explore the significant association between a non-protein coding single nucleotide polymorphism (SNP) rs4977574 of *CDKN2BAS* gene and coronary heart disease (CHD). A total of 590 CHD cases and 482 non-CHD controls were involved in the present association study. A strong association of rs4977574 with CHD was observed in females (genotype: *p* = 0.002; allele: *p* = 0.002, odd ratio (OR) = 1.57, 95% confidential interval (CI) = 1.18–2.08). Moreover, rs4977574 was more likely to be a risk variant of CHD under the recessive model in females (χ^2^ = 10.29, *p* = 0.003, OR = 2.14, 95% CI = 1.31–2.77). A breakdown analysis by age had shown that there was an 87% increased risk of CHD for females younger than 65 years (genotype: χ^2^ = 14.64, degrees of freedom (df) = 2, *p* = 0.0002; allele: χ^2^ = 11.31, df = 1, *p* = 0.0008, OR = 1.87, 95% CI = 1.30–2.70). Similar observation was also found in males younger than 65 years (genotype: χ^2^ = 8.63, df = 2, *p* = 0.04; allele: χ^2^ = 7.55, df = 1, *p* = 0.006, OR = 1.45, 95% CI = 1.11–1.90). *p* values were adjusted by age, sex, smoking, high density lipoprotein cholesterol (HDL-C) and low density lipoprotein cholesterol (LDL-C). Meta-analysis of 23 studies among 36,452 cases and 39,781 controls showed a strong association between rs4977574 and the risk of CHD (*p* < 0.0001, OR = 1.27, 95% CI = 1.22–1.31).

## 1. Introduction

Coronary heart disease (CHD) is the top cause of human death in the United States [1] and Asian countries such as China [2] and Japan [3]. CHD is a complex disease caused by a combination of genetic and environmental factors [4]. Clinical observation has found that atherosclerosis is one of the major pathophysiological mechanisms of CHD [5]. Although atherosclerosis is found to be mainly the result of angiogenesis [6,7], there is a lack of genetic evidence describing the pathogenesis of this disease with greater details.

*CDKN2BAS* gene is a large antisense non–coding RNA, which is differentially expressed in a variety of tissues such as vascular endothelial cells and smooth coronary muscle cells [8,9,10,11]. Non-coding RNAs are involved in the regulation of gene expression through transcriptional and translational control [12]. *CDKN2BAS* expression is shown to be associated with multiple phenotypes [13,14] comprising the risk of coronary disease [15]. Interestingly, *CDKN2BAS* expression has been shown to be regulated by a CHD–associated genetic variant [8]. Regulation of cardiac *CDKN2BAS* expression has been found to play a pivotal role in the development of CHD by altering the dynamics of vascular cell proliferation [16]. Moreover, evidence has shown that *CDKN2BAS* gene variants are associated with CHD [17,18,19]. Genome-wide association study (GWASs) have shown that *CDKN2BAS* gene variants are associated with the risk of multiple diseases comprising type 2 diabetes [20,21], ischemic stroke [22], CHD [17,19], and periodontitis [23] that is prone to develop CHD [23]. *CDKN2BAS* may serve as a biomarker for the risk of atherothrombosis and hemorrhagic stroke, and their recurrence [24].

Common variants of *CDKN2BAS* (including rs4977574) are shown to be associated with myocardial infarction (MI) in European whites [14,17,25,26] and Hispanic population [27]. SNP rs4977574 of *CDKN2BAS* gene is also found to be associated with CHD in European and American–Caucasian [25]. In Chinese population, other *CDKN2BAS* gene variants are shown to be associated with diseases such as type 2 diabetes [28,29], ischemic stroke [30], MI [31], atherothrombotic disease and hemorrhagic stroke [24]. However, there is a lack of investigation for the association between rs4977574 of *CDKN2BAS* gene and CHD in Chinese.

The goal of our study is to test the association between rs4977574 of *CDKN2BAS* gene and CHD in Han Chinese. In addition, a meta-analysis of 11 studies among 36,452 cases and 39,781 controls is also performed to evaluate the contribution of rs4977574 of *CDKN2BAS* gene to the risk of CHD.

## 2. Results

The characteristics of study participants were provided in Table 1. Greater number of older (*p* = 0.0001) or male subjects (*p* = 0.003) were presented in CHD group. Smokers were more frequent in patients with CHD (*p* = 0.018). The high-density lipoprotein cholesterol (HDL-C) levels in the CHD cases were much lower than in the controls (*p* = 0.001). The low density lipoprotein cholesterol (LDL-C) concentration revealed a strong difference between cases and controls (*p* = 0.003). The allele frequency and genotype distributions of rs4977574 were listed in Table 2. Since only genotype distribution of rs4977574 in the female subgroup was consistent with Hardy–Weinberg equilibrium (HWE) (*p* > 0.05), our case-control study was limited in females. As shown in Table 2, there was a significant association of rs4977574 with CHD in females (genotype: *p* = 0.002; allele: *p* = 0.002, odd ratio (OR) = 1.57, 95% confidential interval (CI) = 1.18–2.08). Moreover, rs4977574 was more likely to be a risk variant of CHD under the recessive model in females (Table 3, GA + AA *vs.* GG: χ^2^ = 10.29, *p* = 0.003. OR = 2.14, 95% CI = 1.31–2.77). In addition, we performed a breakdown comparison by age between cases and controls (Table 4). A significant association was observed between rs4977574 and the risk of CHD in females younger than 65 years (genotype: χ^2^ = 14.64, degrees of freedom (df) = 2, *p* = 0.0002; allele: χ^2^ = 11.31, df = 1, *p* = 0.0008, OR = 1.87, 95% CI = 1.30–2.70). No significant difference was found in females aged 65 years or older (*p* > 0.05). Interestingly, the male controls in different age groups showed no deviation of HWE (Table 4, *p* > 0.05). And the similar significant association of rs4977574 with CHD was found in the males younger than 65 years (genotype: χ^2^ = 8.63, df = 2, *p* = 0.04; allele: χ^2^ = 7.55, df = 1, *p* = 0.006, OR = 1.45, 95% CI = 1.11–1.90).

**Table 1:** Epidemiological characteristics in Coronary heart disease (CHD) cases and controls.

Characteristics	Case (590)	Control (482)	*p* Value ^a^
Age (years mean ± SD)	61.73 ± 7.83	58.17 ± 8.79	**0.0001**
Sex (male)	418	254	**0.003**
Smoking (*n*)	144	81	**0.018**
Hypertension (*n*)	172	114	0.152
Diabetes (*n*)	58	33	0.124
Family history (*n*)	28	16	0.281
TG (mmol/L)	2.23 ± 1.02	2.28 ± 1.02	0.441
TC (mmol/L)	4.37 ± 1.08	4.31 ± 0.98	0.425
HDL-C (mmol/L)	1.07 ± 0.25	1.12 ± 0.26	**0.001**
LDL-C (mmol/L)	1.95 ± 1.15	1.75 ± 0.97	**0.003**

^a^: *p* values were adjusted by age and sex; TG: triglycerides; TC: total cholesterol; HDL-C: high density lipoprotein cholesterol; LDL-C: low Density lipoprotein cholesterol.

**Table 2:** Association test of rs4977574 between the CHD cases and non-CHD controls.

Gender	Group	Genotype (Counts)			χ^2^	p (df = 2) ^a^	HWE	Allele (Counts)		χ^2^	p (df = 1)	OR (95% CI)
		**AA**	**AG**	**GG**				**A**	**G**			
All	Case (*n* = 590)	122	305	163			0.36	547	631			
	Control (*n* = 482)	138	267	77	23.41	<0.0001	0.007	543	423	20.30	<0.0001	1.48 (1.25–1.75)
Male	Case (*n* = 418)	86	220	112			0.28	392	444			
	Control (*n* = 254)	70	144	40	12.32	0.014	0.02	284	224	10.27	0.001	1.44 (1.15–1.79)
Female	Case (*n* = 172)	36	85	51			1.00	157	187			
	Control (*n* = 228)	68	123	37	11.4	0.002	0.18	259	197	9.78	0.002	1.57 (1.18–2.08)

^a^: *p* values were adjusted by age, sex, smoking, HDL-C and LDL-C.

**Table 3:** Association between rs4977574 and CHD under the dominant and the recessive models in females ^a^.

Gender	Group	Dominant		χ^2^	*p* (df = 2)	OR (95% CI)	Recessive		χ^2^	*p* (df = 1)	OR (95% CI)
		AA	GG + GA				GA + AA	GG			
Female	Case	36	136				121	51			
	Control	68	160	4.03	0.051	1.59 (0.89–2.62)	191	37	10.29	0.003	2.14 (1.31–2.77)

^a^: *p* values were adjusted by age, sex, smoking, HDL-C and LDL-C.

**Figure 1:**
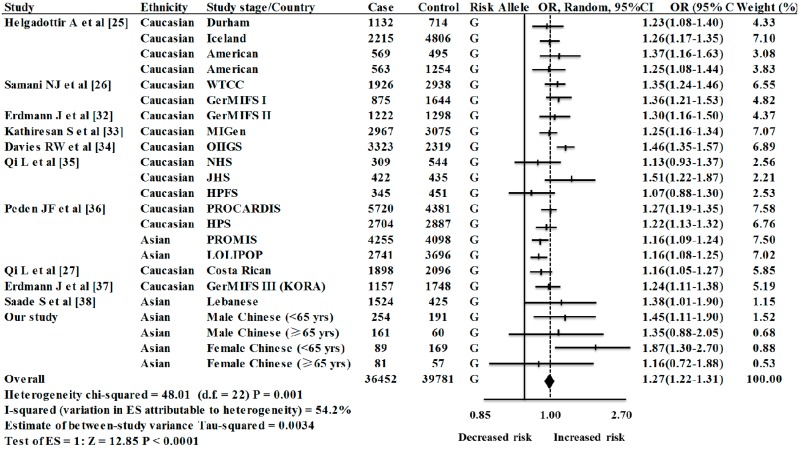
Meta-analysis of eleven studies of rs4977574 and CHD ^a^. a: WTCCC (Wellcome Trust Case Control Consortium); GerMIFSI (German MI Family Study I); GerMIFSII (German MI Family Study II); MIGen (Myocardial Infarction Genetics Consortium); OHGS (Ottawa Heart Genomics Study); NHS (Nurses’ Health Study); JHS (Joslin Heart Study); PROCARDIS (Precocious Coronary Artery Disease); HPS (Heart Protection Study); PROMIS (Pakistan Risk of Myocardial Infarction Study); LOLIPOP (London Life Sciences Prospective Population); GerMIFS III (German MI Family Study (KORA)).

**Figure 2:**
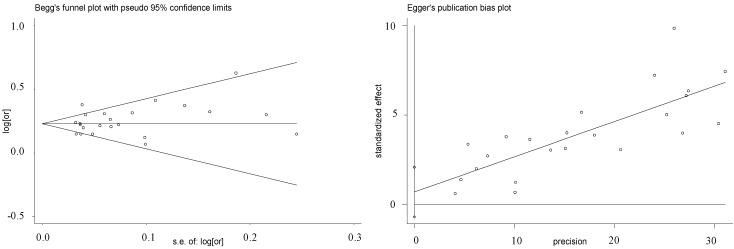
Funnel plot for studies of the association of CHD and rs4977574 ^a^. a: Horizontal axis represents the standard error of log OR; Vertical axis represents the log OR. The “s.e.” denotes standard error.

Our meta-analysis was involved with 23 case-control studies (including four groups in our study) among 36,452 cases and 39,781 controls [25,26,27,32,33,34,35,36,37,38]. Since substantial heterogeneity were observed among the 23 case-control studies (*p* = 0.001, *I*^2^ = 54.2%), random-effect method was applied for the meta-analysis. As shown in Figure 1, there was a significant association between rs4977574 of *CDKN2BAS* gene and the risk of CHD (overall OR = 1.27, 95% CI = 1.22–1.31, random-effect method). No visual publication bias was shown in the Funnel plot and the Egger regression plot (Figure 2). The meta–analysis was involved with case–control studies in Caucasian and Asian populations. Strong association of rs4977574 with CHD were observed in both the Caucasian studies (OR = 1.28, 95% CI = 1.23–1.32, *p* (z) < 0.0001) and the Asian studies (OR = 1.23, 95% CI = 1.13–1.34, *p* (z) < 0.0001). Subgroup study indicated that the ethnicity was likely to be the main source of heterogeneity (Table 5). Specifically, more heterogeneity was found in the Caucasian studies (*I*^2^ = 48.6%, *p* = 0.015), in contrast of moderate heterogeneity in the Asian studies (*I*^2^ = 41.0%, *p* = 0.118).

**Table 4:** Association test in different age subgroups.

Gender	Age	Group	Genotype (Counts)			χ^2^	*p* (df = 2) ^a^	HWE	Allele (Counts)		χ^2^	*p* (df = 1)	OR (95% CI)
		rs4977574	AA	GA	GG				A	G			
Male	<65	Case (*n* = 254)	50	136	68			0.26	236	272			1.45 (1.11–1.90)
		Control (*n* = 191)	53	107	31	8.63	0.04	0.08	213	169	7.55	0.006	
	≥65	Case (*n* = 161)	36	84	41			0.64	156	166			1.35 (0.88–2.05)
		Control (*n* = 60)	15	37	8	3.75	0.30	0.07	67	53	1.91	0.16	
Female	<65	Case (*n* = 89)	18	39	32			0.39	75	103			1.87 (1.30–2.70)
		Control (*n* = 169)	52	91	26	14.64	0.0002	0.20	195	143	11.31	0.0008	
	≥65	Case (*n* = 81)	18	46	17			0.27	82	80			1.16 (0.72–1.88)
		Control (*n* = 57)	15	32	10	0.44	0.63	0.42	62	52	0.38	0.54	

^a^: *p* values were adjusted by age, sex, smoking, HDL-C and LDL-C.

**Table 5:** Heterogeneity test of rs4977574 association studies by subgroup analyses.

Ethnic Group	Studies/Stages (*n*)	OR (95% CI)	Weight (%)	Z	*p*(*z*)	I^2^	P	*τ*^2^	Heterogeneity Statistic
Caucasians	16	1.28 (1.23–1.32)	80.72	13.11	<0.0001	48.6%	0.015	0.0024	29.17
Asians	7	1.23 (1.13–1.34)	19.28	4.74	<0.0001	41.0%	0.118	0.0040	10.16
Overall	23	1.27 (1.22–1.31)	100.00	12.85	<0.0001	54.2%	0.001	0.0034	48.01

## 3. Discussion

The goal of our case-control study was to explore the significant association of *CDKN2BAS* rs4977574 polymorphism with the risk of CHD in Han Chinese. Our results showed that rs4977574 was significantly associated with CHD in females (genotype *p* = 0.002; allele *p* = 0.002, OR = 1.57, 95% CI = 1.18–2.08). Interestingly, this SNP was more likely to increase the risk of CHD under the recessive model in females (GA + AA *vs.* GG: χ^2^ = 10.29, *p* = 0.003, OR = 2.14, 95% CI = 1.31–2.77). In addition, we also found that rs4977574 might have an 87% and 45% increased risk of CHD in females and males younger than 65 years, respectively. Our meta–analysis among 36,452 cases and 39,781 controls concluded that rs4977574 of *CDKN2BAS* gene contributed to the risk of CHD in both Asian and Caucasian populations, although there was substantial heterogeneity among the involved studies (*I*^2^ = 54.2%).

SNP rs4977574 on chromosome 9p21.3 is located in *CDKN2BAS* (also known as *ANRIL*). This region has been considered as the most widely and consistently replicated risk locus for CHD and MI [18,39]. The function of *CDKN2BAS* is unknown, but the *CDKN2BAS* transcript level shows bold correlation with the severity of atherosclerosis [14]. The modulation of *CDKN2BAS* gene expression mediates susceptibility to several important human diseases such as CHD and cancer [15]. The 9p21.3 risk allele in CHD is associated with altered expression of *CDKN2BAS* gene in blood [8]. Evidences have demonstrated that *CDKN2BAS* is a new susceptibility gene for the risk of CHD [14,36,40].

Sex differences have been proved in the prevalence and clinical outcomes of subclinical peripheral artery disease (PAD) [41], and the females have a higher prevalence of subclinical PAD [41]. The gender difference can be regarded as a genetic risk profile for cardiovascular disease (CVD) [42] such as CHD [43]. The genetic risk loci for CVD are more readily detectable in females, while the males they are more confounded by environmental or lifestyle risk factors [44]. In this study, significant departure from HWE is observed in the male controls, although male controls in different age subgroups are shown with no deviation of HWE. Since we didn’t observe these phenomena for other variants using the same sample sets [45,46,47,48,49,50], the chance of genotyping errors and DNA contamination is minimal to cause the departure of HWE for the male controls. We speculate that it may be due to the comparatively small sample size for the male controls (*n* = 254 *vs.* 482 in male cases). Multiple-center collaboration for a better coverage of the Han Chinese population in Ningbo city is warranted for the association test of this variant in the future.

Early-onset CHD is more suitable for genetic analysis [51], because heritability of the early-onset CHD is higher than that of the late-onset one [52]. Genetic polymorphisms may play an important role in the pathogenesis of early onset CHD [53]. Gongqing Shen *et al*. [54] reported that the polymorphisms of Low-density lipoprotein receptor-related protein 8 (*LRP8*) were risk factor of CHD. The risk haplotype TACGC in *LRP8* existed only in patients with familial and early onset CHD [54]. Monika Rać *et al*. [55] confirmed that the CD36 gene polymorphisms were involved with echo and electrocardiographic parameters in patients with early onset CHD. Alstrom syndrome 1 (*ALMS1*) gene polymorphisms were shown to be significantly associated with early-onset MI in both Japanese and Korean populations [56]. Some evidence has reported on associations of variant in chromosome 9p21 with early onset CHD in different populations [57,58,59,60]. In the present study, rs4977574 at the 9p21 locus is found to be associated with the 87% and 45% increased risk of CHD in both females and males younger than 65, respectively. Our results may provide new clues to predict the risk of early onset CHD, and may help to elaborate the mechanisms by which *CDKN2BAS* exerts its effects on the risk of CHD.

The sample size is comparatively small for the breakdown analysis by age and gender. Although the sample size may not be the optimal, our power calculation for male and female (<65 year) suggests that our study has a 78.2% and 92.1% power to detect a relative risk at a significant level of 0.05 which should be sufficient to describe a tendency to guide clinical practice. Since all the subgroups meet with HWE, we integrate the four subgroups into the current meta-analysis. For the meta-analysis among a total of 36,452 cases and 39,781 controls, our results show that rs4977574-G can increase 27% of CHD risk (*p* < 0.0001, OR = 1.27, 95% CI = 1.22–1.31). This agrees with the previous meta-analysis [36] with only four studies of rs4977574 in Europeans and South Asians. Our meta-analysis has included 23 case-control study stages that have 19 more datasets than the previous meta-analysis [36]. Our subgroup analysis of 23 studies shows that there is significant heterogeneity in Caucasians (Table 5). This may be explained by the existence of hidden gene structure in ethnic composition and various experimental designs among different studies. HapMap International Project has shown there are huge ethnic differences of rs4977574-G allele frequency between the African populations (7.5%–21%) and the rest populations, including Asians (45%–50%) and Caucasians (46%–50%) in the North America and Europe. Future investigation of this important variant in other populations such as Africans is intriguing.

## 4. Experimental Section

### 4.1. Sample Collection

A total of 590 CHD cases (418 males and 172 females; mean age: 61.73 ± 7.83 years) and 482 non-CHD controls (254 males and 228 females; mean age: 58.17 ± 8.79 years) were recruited between May 2008 and April 2012 from the Lihuili Hospital in Ningbo city of Zhejiang province, China. Each of the CHD cases had stenosis greater than 50% in one or more major coronary arteries [61] or a history of prior angioplasty or coronary artery bypass surgery. Non-CHD controls had stenosis less than 50% in any of the major coronary arteries, and did not have any atherosclerotic vascular disease. All the samples were Han Chinese originated from Ningbo city in the Eastern China. All the participants had been diagnosed through the standardized coronary angiography according to the Seldinger’s method [62], and judged by two or three independent cardiologists. All individuals were excluded from congenital heart disease, cardiomyopathy, and severe liver or kidney diseases. Blood samples were collected and treated by the same investigators. This study was approved by the Ethical Committee of Lihuili Hospital (project identification code: 2008032114), and all subjects were informed with written consent.

### 4.2. Biochemical Analysis

Blood samples were obtained after a 12 h overnight fast from subjects using 3.2% citrate sodium-treated tubes. Plasma levels of triglycerides (TG), total cholesterol (TC), and HDL-C, LDL-C were enzymatically measured using standard methods on an Olympus AU2700 automatic analyzer (Olympus, Tokyo, Japan).

### 4.3. SNP Genotyping

Genomic DNA was isolated from whole blood by the conventional phenol/chloroform extraction method and was stored in 200 μL of TE buffer. DNA concentration was quantified using the Biophotometer plus (Eppendorf, Hamburg, Germany) with the manufacturerʼs protocol. The primers for the Polymerase Chain Reaction (PCR) were as followed: forward primer, 5'-ACGTTGGATGGGACATCTTTTGTTAGAGTG-3'; reverse primer, 5'-ACGTTGGATGGTTTGCTTTCAGGGTACATC-3'; extension primer, 5'-CCCGACATCAAATGCATTCTATAGC-3'. DNA amplification was performed on the ABI Geneamp PCR System 9700 Dual 384-Well Sample Block Module (Applied Biosystems, Foster City, CA, USA). PCR cycling program included a 15 s initial denaturation stage at 94 °C, followed by 45 cycles of denaturation for 20 s at 94 °C, annealing for 30 s at 56 °C, and extension at 72 °C for 1 min, and a final extension for 3 min at 72 °C. Allele specific primer extension was performed on the Sequenom MassARRAY iPLEX platform according to the manufacturer’s instructions [63]. To verify the repeatability and stability of experiment, 18 random samples and 18 control samples (including 9 negative and 9 positive controls) were used for quality control.

### 4.4. Retrieval of Published Studies and Selection of Studies for the Meta-Analysis

We examined all studies by a search of the papers published on the electronic databases (PubMed (Bethesda, Maryland, USA), Web of Science (Stamford, CT, USA) and the Cochrane Library (Oxford, Oxfordshire, UK)) from 2009 to 2012. Various combinations of keywords were used to the following search terms, such as “coronary heart disease” or “coronary artery disease” or “myocardial infarction” combined with “*CDKN2BAS*”, “*CDKN2B*–*AS1*” or “*ANRIL*”, “polymorphism” and “genetic association”. Interested information was selected after reading the full text articles. Other articles from the reference list on the retrieved and previous meta–analysis of this subject were evaluated based on the potential relevance. In addition, the authors of the retrieved papers were contacted directly for any additional and unpublished data. The inclusion criteria for the study were as follows: (1) case-control or cohort study; (2) the studies for which odds ratios (ORs) and 95% CIs were given, or could be calculated on the basis of genotype and allele frequencies. A random-effect model was applied when heterogeneity was detected (*I*^2^ > 50%) [64]. Data extraction was enforced alone by two or three reviewers on the basis of a standard method. Consensus data were established though discussion in case of controversy. In the current meta–analysis, the extracted data included the first author’s name, publication year, country, ethnic population, design of study, total number of cases and controls, OR and 95% CI.

### 4.5. Statistical Analyses

*T*-test was applied for the association of CHD with continuous variables including age, TG, TC, HDL-C and LDL-C. Pearson chi-square or Fisher exact test was used for categorical variables including sex, smoking, hypertension, diabetes and family history. Multivariable logistic regression analysis was used to detect association of the parametric and nonparametric phenotypes with genotypes, respectively. The departures of HWE of the genotypes were analyzed by the Arlequin program (version 3.5, Bern, Switzerland) [65]. Comparison of the genotype and allele frequencies between cases and controls was determined by the CLUMP16 software (Denmark Hill, London, UK) with 10,000 Monte Carlo simulations [66]. ORs and 95% CIs were calculated using the PASW Statistics 18.0 software (SPSS, Inc., Somers, NY, USA) [67]. The power of the study was evaluated by the Power and Sample Size Calculation software (v3.0.43, TN, Nashville, TN, USA) [68]. Meta-analysis was performed by the REVMAN software (version 5.0, Cochrane Collaboration, Oxford, UK) and the Stata software (version 11.0, Stata Corporation, College Station, TX, USA) [69]. The publication bias was visualized by Funnel plots and Egger regression plot [70]. According to the heterogeneity level of meta-analysis, either fixed-effect or random-effect method was used to assess the combined ORs along with their 95% CIs. A two-tailed *p* value <0.05 was considered to be significant.

## 5. Conclusions

Our case-control study has identified a significant association of rs4977574 with the risk of CHD under a recessive inheritance model in females. Meta-analysis of 23 studies among 36,452 cases and 39,781 controls has established rs4977574 as a risk factor of CHD in multiple populations including Asians and Caucasians.
